# Establishment of a Stable Cell Line Expressing Green Fluorescence Protein-fused Hypoxia Inducible Factor-1α for Assessment of Carcinogenicity of Chemical Toxicants

**DOI:** 10.5487/TR.2009.25.4.189

**Published:** 2009-12-30

**Authors:** Sung-Hye Kim, Hee-won Seo, Min-Ho Lee, Jin-Ho Chung, Byung Hoon Lee, Mi-Ock Lee

**Affiliations:** 14 College of Pharmacy, Seoul National University, Seoul, 151-742 Korea; 24 Bio-MAX Institute, Seoul National University, Seoul, 151-742 Korea; 34 College of Pharmacy and Bio-MAX Institute, Seoul National University, San 56-1 Sillim-dong, Gwanak-gu, Seoul, 151-742 Korea

**Keywords:** GFP-HIF-1α stable cell line, Chemical toxicants, Carcinogenicity

## Abstract

Hypoxia inducible factor 1α (HIF-1α) is a potential marker of carcicnogenesis since it is overexpresssed in many human cancers such as brain, breast, and uterus, and its role has implicated in tumor cell growth and metastasis. In this study, we established a stable cell line that express green fluorescence protein (GFP)-fused hypoxia inducible factor 1α (HIF-1α) and evaluated the potential use of this cell line for assessment of carcinogenicity of chemical toxicants. Western blot analysis as well as fluorescence measurements showed that protein-level of GFP-HIF-1α was significantly enhanced in a dose-dependent manner upon treatment of hypoxia mimicking agents such as dexferrioxamine and CoCl_2_. Well-Known tumor promoters such as mitomycin and methyl methane-sulfonate. significantly induced the fluorescence intensity of GFP-HIF-1α, whereas the known negative controls such as *o*-anthranilic acid and benzethonium chloride, did not. These results indicate that HIF-1α could be a biological parameter for detection of tumor initiators/promoters and suggest that the GFP-HIF-1α cell line is a useful system for screening of carcinogenic toxicants.

## INTRODUCTION

Carcinogenesis is typically a multistage nature consisting of initiation, promotion, and progression. Initiation stage is thought to induce mutations in the critical genes which confer a selective growth advantage to specific cells. Promotion stage induces the tumor formation and a potential progression to malignancy by occurring the additional genetics or epigenetic changes (Trosko, 2001; Humble *et al*., 2005). Though many *in vitro* assays have been developed with aim of detecting the carcinogenesis of chemical compounds, few of that satisfied in model for carcinogenesis testing.

Hypoxia inducible factor-1 (HIF-1) plays an essential role in cellular and systemic oxygen homeostasis. HIF-1 is a key transcriptional activator that is induced in hypoxia condition and regulates cell adaptation and survival to hypoxia. HIF-1 is a heterodimer that consists of a HIF-1α subunit and a constitutively expressed HIF-1β subunit (Machon *et al*., 2001; Semenza, 2003). In normal condition, HIF-1α is rapidly degraded through the ubiquitin-proteasome pathway, while it is stabilized under hypoxia condition (Huang *et al*., 1998). More than 60 putative direct HIF-1 target genes have been identified which are particularly involved in crucial aspects of cancer biology, including cancer angiogenesis, glucose metabolism, cell proliferation/survival and invasion/metastasis (Semenza 2003; Lee *et al*., 2004). Recently, many studies revealed that HIF-1α is overexpresssed in many human cancers such as brain, breast, lung, cervix, ovary and uterus (Zagzag *et al*., 2000; Biner *et al*., 2001; Schindl *et al*., 2002; Burri *et al*., 2003; Swinson *et al*., 2004). The association between HIF-1 and cancer was observed among tumors from most of patients (Zhong *et al*., 1999). Together, these results indicate that HIF-1α is a potential marker of carcicnogenesis.

Previously we have developed a stable cell line of which chromosome containing a reporter encoding hypoxia response element that express luciferase in response to transcriptional activation of HIF-1. The benefit of this system included a high sensitivity and a good correlation with *in vivo* test (Hong *et al*., 2007). In the present investigation, we established a new stable cell line that express green fluorescence protein (GFP)-

fused HIF-1α, to make an advance in the HIF-1 monitoring system in the aspects of handling process and experimental cost. The newly developed method could be easily applicable to an alternative screening method for tumor promoters, since this method requires only a simple process of fluorescence measurements.

## MATERIALS AND METHODS

***Cells and chemicals.*** The human cervical carcinoma cell line, HeLa (ATCC CCL-2) was obtained from the American Type Culture Collection. Cells were maintained in Dulbecco's modified eagle's medium containing 10% fetal bovine serum at 37°C in a 5% $CO_2$/95% air incubator. Hypoxia was induced chemically by treating cells with $CoCl_2$ (Sigma, St. Louis, MO) or dexferrioxamine (DFO) (Calbiochem, San Diego, CA). *o*-Anthranilic acid, benzethonium chloride, mitomycin (MMC), methyl methanesulfonate (MMS), methoxychlor, N-(1-naphthyl) ethylenediamine dichloride, griseofulvin and thioacetamide were purchased from Sigma.

***Establishment of the GFP-HIF-1α stable cell line.*** HeLa cells were seeded in 60-$cm^2$ dishes ($1 \times 10^5$ cells/dish) and incubated overnight. The cells were transfected with a 0.1 μg GFP-HIF-1α expression vector and pcDNA3.1/neo which is neomycin resistant gene using LipofectaminePlus (Invitrogen, San Diego, CA) according to the manufacturer's instructions. Cells were cultured for 6 weeks in the presence of 800 μg/ml G418 and survival colonies are selected and maintained in media containing 600 μg/ml G418.

***Fluorescence measurements.*** HeLa cells were seeded in 12-well plates ($1 \times 10^4$ cells/plate) or 24-well plates ($1 \times 10^5$ cells/plate) and incubated overnight. At 24 h after the treatment with DFO, $CoCl_2$, and other chemicals known as tumor promoters, fluorescence was detected by fluorescence microscopy.

***Quantitative analysis for fluorescence of GFP-HIF-1α.*** HeLa cells were seeded in black 96-well plates ($6 \times 10^3$ cells/plate) and incubated overnight. At 24 h after treatment of chemicals, the media were changed to phosphate buffered saline. Fluorescence intensity of plates was measured using Softmax software of High Throughput Screening (BiomekFX, Backman Coulter) with excitation at 488 nm and emission of 535 nm using bottom read. The quantitative data were shown as mean value of 10 replicates. MMC, MMS, griseofulvin and thioacetamide are known tumor iniciator/promoters whereas *o*-anthranilic acid, benzethonium chloride, methoxychlor and N-(1-naphthyl) ethylenediamine dichloride are negative controls (Haseman *et al.*, 1987; Spalding *et al.*, 1999).

***Western blotting.*** Western blotting was performed as previously described using specific antibodies against GFP (Santa Cruz Biotechnology) and α-tubulin (Calbiochem) (Yoo *et al.*, 2004).

***Statistical analysis.*** Experimental values were expressed as mean ± SEM. The significance of differences were determined by student's *t*-test and expressed as a probability value. Mean differences were considered to be significant when $P < 0.05$.

## RESULTS AND DISCUSSION

Many *in vivo* and *in vitro* assays have been developed for detection and characterization of chemical toxicants and for detection and prevention of human cancer (Tennant *et al.*, 1987, 1988). Among these assays, in vitro transformation assay is not only regarded as a major method of the screen of potential carcinogens promoters, but is also an important approach for the study multistage carcinogenesis (Berwald and Sachs, 1963; Breheny *et al.*, 2005). However, this assay has not been exploited, since it requires a long period of cultivation, low transformation frequency, high cost, and high effort (Grover *et al.*, 1971; Fang *et al.*, 2001). Though this method was recently modified to make it more convenient, low transformation frequency is still not suitable for performing studies (Tsuchiya and Umeda, 1997; Custer *et al.*, 2000). We have concentrated efforts on development of *in vitro* system for rapid detection of carcinogenic agents. We have previously developed a stable cell line of which chromosome containing a reporter encoding hypoxia response element that express luciferase in response to transcriptional activation of HIF-1 (Hong *et al.*, 2007). The benefit of this system included sensitivity and a good correlation with the results obtained from *in vivo* test (Hong *et al.*, 2007). Here, we established a new stable cell line expressing GFP-HIF-1α, to make an advance in the HIF-1 monitoring system in the aspects of handling process and experimental cost.

To establish the stable cell line expressing GFP-fused HIF-1α, the human cervical carcinoma cell line, HeLa cells were co-transfected with a plasmid encoding GFP-HIF-1α and a vector encoding neomycin resistant gene, and cultured for 6 weeks in the presence of G418. Four survival colonies were obtained and one which responded sensitively to DFO was selected for further

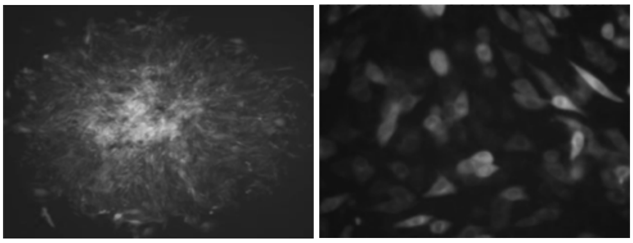

**Fig. 1.** Establishment of a stable cell line expressing GFP-HIF-1α. HeLa cells were transfected with a 0.1 μg GFP-HIF-1α expression vector and pcDNA3.1/neo which is neomycin resistant gene using LipofectaminePlus according to the manufacturer's instructions. Cells were cultured for 6 weeks in the presence of 800 μg/ml G418 and survival colonies are selected and maintained in media containing 600 μg/ml G418. Representative images for a colony formed (left, × 40) and stable cells expressing GFP-HIF-1α (right, × 400) taken by fluorescence microscopy were shown.

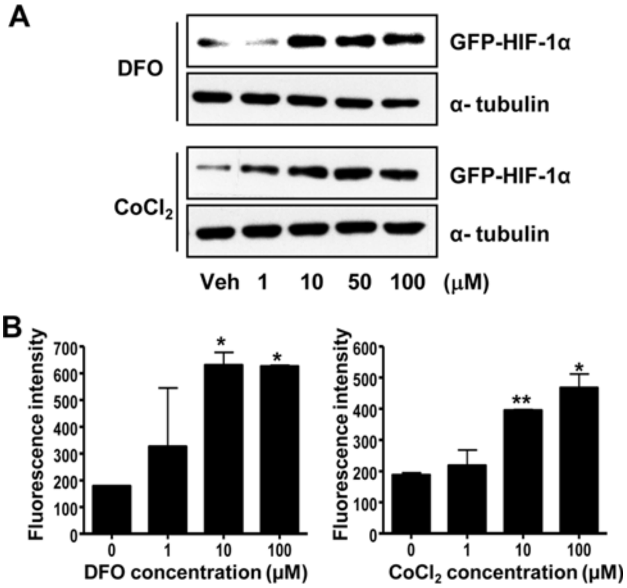

**Fig. 3.** Induction of GFP-HIF-1α protein after treatment with hypoxia mimicking chemicals in the GFP-HIF-1α stable cell line. (A) The GFP-HIF-1α stable cells were seeded in 60-mm$^2$ dishes (4 × $10^5$ cells/dish) and incubated overnight. At 24 h after the treatment with indicated concentrations of DFO and $CoCl_2$, whole-cell lysates were prepared and the expression of proteins was analyzed using anti-GFP antibody by western blotting. (B) GFP-HIF-1α stable cells were seeded in the block 96-well plates (6 × $10^3$ cells/plate) and incubated overnight. At 24 h after the treatment, fluorescence was measured using Softmax software of HTS. Each bar represents the mean ± SEM of at least three experiments. *$p < 0.05$ and **$p < 0.01$.

study (data not shown). Fig. 1 shows the colony formed and the established cell line that expressed GFP-HIF-1α protein. The response to hypoxia was examined after treatment of DFO or $CoCl_2$. After 24 h of treatment, the fluorescence of GFP-HIF-1α was significantly increased in a dose-dependent manner (Fig. 2), indicating that HIF-1α protein was stabilized in the presence of hypoxic stress. To confirm the increased expression of GFP-HIF-1. protein, we analyzed protein-level of GFP-HIF-1α by western blot analysis. As shown in Fig. 3A, the amount of GFP-HIF-1α protein was increased in a dose-dependent manner. To quantitate fluorescence of GFP-HIF-1α, the stable cells were seeded in 96-wll plate, treated with DFO or $CoCl_2$ and the fluorescence of the cells were directly measured using Softmax software of high throughput screening. Consistent with the western blotting results, fluorescence of the cells was increased dose-dependently (Fig. 3B). These results indicate that a stable cell line which expressed GFP-HIF-1α was successfully established.

Next, we studied whether our stable cell line could serve as a useful system for assessment of carcinogenicity of chemical toxicants. We examined the expression of GFP-HIF-1α after treatment of four known carcinogens, *i.e.*, MMC, MMS, griseofulvin and thiaceta-

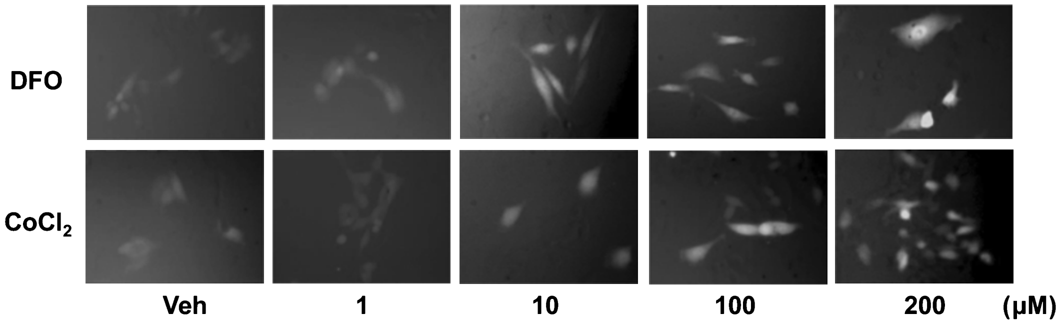

**Fig. 2.** Fluorescence of the GFP-HIF-1α stable cells induced by hypoxia mimicking agents. The GFP-HIF-1α stable cells were seeded in 24-well plates (1 × $10^5$ cells/plate) and incubated overnight. At 24 hours after the treatment with indicated concentrations of DFO (A) and $CoCl_2$ (B), expression of GFP-HIF-1α was detected using fluorescence microscopy (× 400). Representative images obtained from at least three experiments were shown.

mide. MMC is a well-known mutagenic and clastogenic agent which induces base substitution, sister chromatid exchange, chromosomal aberration, and micronuclei formation *in vivo* and *in vitro* (Concepcion Becerril *et al.*, 1999; Tomasz *et al.*, 1974). MMS is a highly toxic DNA-alkylating agent, which methylates the DNA bases and causes DNA damage leading to strand breaks, chromosome breaks, micronucli formation, and cell death (Wyatt *et al.*, 2006; Lee *et al.*, 2007). Griseofulvin is a mycotoxin produced by various species of *Penicillium* and it has been used as a potent antifungal agent against dermatophyroses. Griseofulvin promoted skin tumor development in mice when given orally after methylcholanthrene initiation and affected a promotion stage of carcinogenesis (Vesselinovitch and Mihailovich, 1968; Labay *et al.*, 2001). Thioacetamide, originally used as a fungicide, is a potent hepatotoxin. Thioacetamide stimulates DNA synthesis and mitosis in the liver of rats at doses that produce limited necrosis (Mangipudy *et al.*, 1996, Chilakapati *et al.*, 2005). After 24 h of treatment with these toxicants, the fluorescence of GFP-HIF-1$\alpha$ was significantly increased at the dose ranges that are employed for carcinogenicity tests. The expression level of GFP-HIF-1$\alpha$ was enhanced 5 to 6 times in the presence of these toxicants (Fig. 4). In contrast, after 24 h of treatment with four known negative controls, the fluorescence of GFP-HIF-1$\alpha$ did not increased. These results demonstrate a good predictivity for carcinogeneicity and validate our GFP-HIF-1$\alpha$ stable cell line as a potential screening method. Since the newly developed GFP-HIF-1$\alpha$ stable cell line system could provide significant improvement in handling process and experimental costs, it is easily applicable to an alternative screening method for carcinogenic toxicants.

## ACKNOWLEDGMENTS

This study was supported by grants from the Korea Food and Drug Administration for Studies on Standardization of Herbal Medicine (2009).

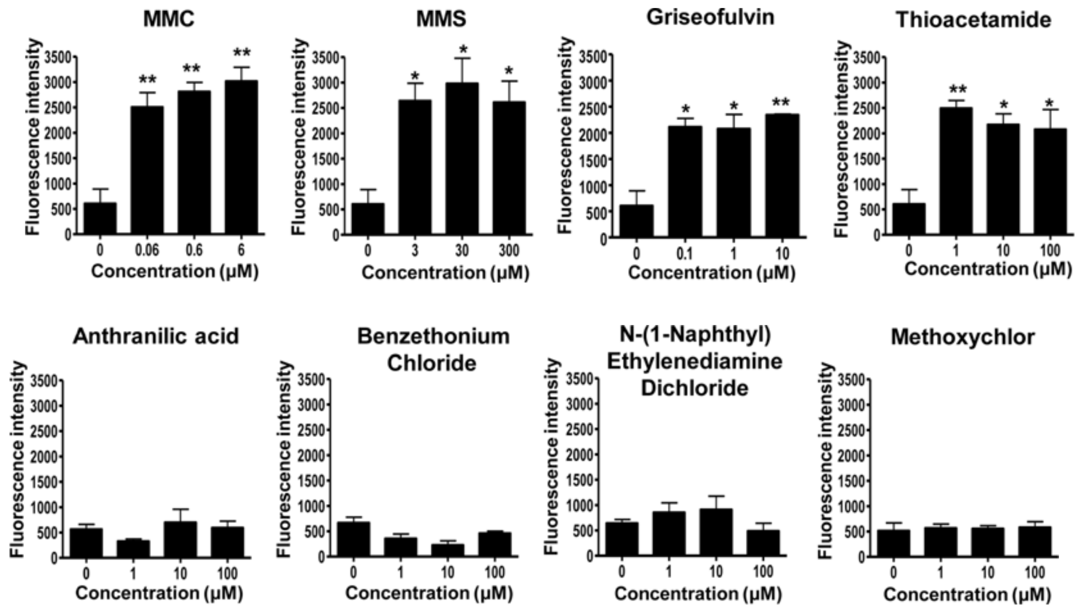

**Fig. 4.** Quantitative analysis of expression of GFP-HIF-1$\alpha$ after treatment with chemical toxicants. GFP-HIF-1$\alpha$ stable cells were seeded in the block 96-well plates (6 × 10 cells/plate) and incubated overnight. At 24 h after treatment of the indicated chemicals, fluorescence was measured using Softmax software of HTS. Each bar represents the mean ± SEM of at least three experiments. *p < 0.05 and **p < 0.01.

Zagzag. D., Zhong, H., Scalzitti, J.M., Laughner, E., Simons, J.W. and Semenza, G. L. (2000). Expression of hypoxia-inducible factor 1$\alpha$ in brain tumors. *Cancer*, **88**, 2606-2618.

Zhong., H., De Marzo, A.M., Laughner, E., Lim, M., Hilton, D.A., Zagzag D., Buechler, P., Isaacs, W.B., Semenza, G.L. and Simons, J.W. (1999). Overexpression of hypoxiainducible factor 1$\alpha$ in commom human cancers and their metastases. *Cancer Res.*, **835**, 5830-5835.
